# Cardioprotective effects of preconditioning exercise in the female tumor bearing mouse

**DOI:** 10.3389/fcell.2022.950479

**Published:** 2022-12-01

**Authors:** Traci L. Parry, Louisa Tichy, Jason T. Brantley

**Affiliations:** Department of Kinesiology, University of North Carolina Greensboro, Greensboro, NC, United States

**Keywords:** heart, cancer, muscle wasting, exercise, autophagy

## Abstract

Cancer cachexia, a metabolic wasting syndrome, affects up to 80% of cancer patients and leads to the death in up to 20% of cancer patients. While research is growing in the field, there are still no clear diagnostic criteria and cancer cachexia remains an untreated condition. Aerobic exercise has been shown to positively impact cachexia by slowing its development and attenuating muscle loss. The most effective timing, duration, and intensity of exercise as a preventative and protective measure against cancer cachexia remains questionable. Therefore, the purpose of this study was to examine the effects of preconditioning exercise as a protective measure for tumor-mediated muscle wasting. Female LC3 Tg+ and wildtype mice were randomly separated into four groups, sedentary non-tumor bearing (SED + NT), sedentary tumor bearing (SED + T), treadmill exercise non-tumor bearing (TM + NT), and treadmill exercise tumor bearing (TM + T). Mice underwent an 8-week treadmill exercise training protocol (TM) or remained sedentary (SED). Next, mice were implanted with tumor cells (T group; 5 × 10^5^ Lewis Lung Carcinoma cells in flank) or remained non-tumor (NT) for 4 weeks. Tumor bearing resulted in a significant decline in cardiac function. SED + T showed a significant decrease in fractional shortening (*p* < 0.05) when compared to the other groups. This coincided with an increase in beclin-1 and MyD88 protein expression and decrease in p-FOXO1 (inactivated) protein expression in SED + T mice. Interestingly, preconditioning exercise (exercise prior to tumor bearing) appeared to preserve cardiac function (TM + T not significantly different than SED + NT). Exercise-mediated cardioprotection also coincided with abolished beclin-1 and MyD88 signaling that was not significantly elevated in TM + T mice. Additionally, TM resulted in a 22-fold decrease in estimated tumor volume (*p* < 0.05) and a 45% decrease in tumor mass (*p* < 0.05) compared to SED tumors. The data indicate potential cardioprotective effects of preconditioning exercise on preserving cardiac structure and function, as well as regulating autophagic (beclin-1), inflammatory (TGF-β and MyD88), and atrophy (p-FOXO1) pathways during tumor bearing. Preconditioning exercise may be an effective and accessible treatment intervention for early-stage cancer survivors. This data is crucial in identifying the significance of exercise and the timing of exercise as a protective measure against the detrimental effects of cancer cachexia.

## Introduction

Cancer remains one of the leading causes of death worldwide. In the US, there are currently 13.7 million people with cancer and another 1.7 million will receive a diagnosis this year (American Cancer Society). Up to 80% of cancer patients will develop cancer cachexia, a debilitating wasting disorder caused by underlying metabolic derangement that leads to poor treatment responses and markedly decreased survival (Kumar et al., 2010; Fearon et al., 2011; Argiles et al., 2014). There is mounting evidence that cancer cachexia can cause cardiac wasting and dysfunction in addition to proteolysis and atrophy of skeletal muscle and wasting of fat tissue (Kazemi-Bajestani et al., 2014; Couch et al., 2015; Murphy, 2016).

The autophagy lysosome system is a bulk protein degradation system that constitutes an important role in tissues like the heart that are not capable of regeneration. Autophagy system has been shown to play a role in muscle wasting (Sandri, 2013) and in cancer cachexia (Penna et al., 2013; Aversa et al., 2016). Since autophagy plays a role in cardiac metabolism (Parry and Willis, 2016), autophagy likely contributes to metabolic changes that occur during cancer cachexia and tumor-mediated muscle wasting.

Exercise and physical activity reduces the risk of all-cause mortality (Zhao et al., 2020) and can lower the risk of several types of cancer (Nilsson et al., 2019). While the cardioprotective effects of exercise are well documented (Quindry et al., 2007; Kavazis et al., 2008; Powers et al., 2014; Parry et al., 2018), less is known about the overall protection of preconditioned exercise–exercise implemented prior to a challenge or intervention. Given the ability of exercise to lower risk of chronic illness and disease, understanding the benefits of the timing of exercise remains critical. Therefore, the purpose of this study was to determine the possible protective role of exercise preconditioning against tumor-mediated cardiac dysfunction and wasting, and to determine the role of exercise preconditioning on tumor growth.

## Methods

### Experimental design

The study was performed on 6 month old LC3 transgenic (LC3 Tg+) mice (C57BL/6-Tg (CAG-RFP/EGFP/Map1lc3b)1Hill/J; Jackson Laboratory strain #027139) and sibling/age matched wildtype (WT) controls. All groups (outline below) are balanced 50:50 with WT:LC3 Tg + sibling matched mice which accounts for the slight variations in animals per group. All mice were bred onsite in a temperature-controlled animal facility with a 12:12 light-dark cycle, and internally maintained from breeder rotation. During the length of the protocol, mice were housed in standard mouse cages and provided with distilled water and standard rodent chow *ad libitum*. All procedures were approved by the University of North Carolina at Greensboro’s Institutional Animal Care and Use Committee and complied with the Animal Welfare Act guidelines. Mice were randomly selected and separated into four groups: sedentary non-tumor bearing (SED + NT; n = 9), sedentary tumor bearing (SED + T; n = 8), treadmill trained non-tumor bearing (TM + NT; n = 7), and treadmill trained tumor bearing (TM + T; n = 9). Animals either underwent a preconditioning treadmill exercise protocol (TM groups) or remained sedentary (SED groups) for 8 weeks. After the 8-week activity protocol, mice were either inoculated with tumor cells (T groups; 5 × 10^5^ LLC cells in flank) or remained non-tumor (NT groups) for an additional 4 weeks. All animals were sacrificed at the end of the 12-week protocol. Cardiac muscle function was assessed by echocardiography, and tumor size was measured with calipers. Heart and tumor tissues were collected, weighed, and either flash frozen in liquid nitrogen or embedded in optimal cutting temperature gel (OCT) and frozen on dry ice for subsequent biochemical analysis.

### Tumor model

Lewis Lung Carcinoma (LLC), a mouse lung tumor cell line (American Type Culture Collection, CRL-1642; Manassas, VA, United States ) was used to grow a subcutaneous tumor in the flank of the tumor-bearing mice. Cells were grown in Dulbecco’s Modified Eagle Medium (DMEM; ATCC #30–2002; Manassas, VA, United States ) supplemented with 10% fetal bovine serum, in an incubator that was set to 5% CO_2_ and 37°C. At least 24 h after the end of the activity protocol, mice were inoculated with a concentration of 5 × 10^5^ LLC cells in 100 μL of sterile phosphate buffered saline (PBS; Gibco, Massachusetts, United States ) subcutaneously in the left flank. Body weight, body conditioning, and tumor measurements were taken three times a week. Tumor length, width, and thickness were measured three times per week by using a Vernier caliper. Tumor measurements were used to estimate tumor mass, relative tumor mass, and tumor volume using the following formulas:

tumor mass(g)=0.79768+(0.000456*length*width*thickness of tumor in mm) (1)

relative tumor mass=estimated tumor mass/(total body mass−estimated tumor mass) (2)

tumor volume (mm3 )=(a∗b^2)/2 (3)

If estimated tumor mass exceeded 25% of body mass, percent loss of tumor free body mass exceeded 25% of starting mass, tumor became ulcerated, or the animal received a body conditioning score ≤2, mice were to be euthanized and removed from the study. It should be noted that two mice met this criterion within 4 days of the end of the study and were thus removed from the study (one from the SED + T group, one from the TM + T group). The present study reflects only animals that reached the end of the 4-week tumor bearing period (end of 12-week study timepoint).

### Treadmill protocol

Mice were separated into preconditioning exercised and sedentary groups. The preconditioning treadmill exercise protocol lasted for 8 weeks and was progressive in nature, reaching an intensity of approximately 65%VO_2_max of C57/BL6 mouse strain during the last 2 weeks of the protocol. Treadmill trained groups underwent an acclimation period, consisting of treadmill running at low speed and gradually increasing speed to 15 m/min for a total of 20 min per session on 3 days during the week prior to the start of the preconditioning protocol. Throughout the preconditioning protocol, mice ran for 45–60 min on 5 days each week with a consistent speed of 15 m/min for each session. During week 1 and 2, treadmill was set at an incline of 0° for 45min. Duration was increased to 60min per session starting in week three until the end of the protocol. Incline was increased to 2° during week 5 and 6, and finally increased to 4° during week seven and 8. Treadmill exercise was performed during the light cycle, between 6:00 and 12:00.

### Echocardiography

At baseline and sacrifice timepoints, mice were loosely restrained in a supine position and hair was removed from the chest by means of a depilatory agent. Warm transduction jelly was applied to the chest. Two-dimensional M-mode echocardiography (GE Vivid seven Dimension) was performed in the parasternal long-axis view at the level of the papillary muscle. Measurements represented average of three cardiac cycles from each animal. Posterior and anterior wall thickness was measured as distance from epicardial to endocardial leading edges. Left ventricular diameters were measured and cardiac function was assessed by fractional shortening: (FS)%=[(LV end diastolic diameter−LV end systolic diameter)/ LV end systolic diameter]∗100 .

### Sacrifice and tissue harvest

All mice were sacrificed at the end of the 12-week protocol *via* isoflurane anesthesia, followed by cervical dislocation. Muscles were harvested into 1.5 ml internally threaded cryogenic storage vials and immediately flash frozen in liquid nitrogen. Cardiac muscles of LC3 Tg + mice were excised and immediately placed in frozen tissue trays, embedded with OCT Compound (Tissue-Trek, Sakura Finetek United States , Inc., Torrance, CA, United States ) and frozen immediately on dry ice (<1min). All tissues were stored at -80°C until further analyses.

### Confocal fluorescent analysis of autophagic puncta

In OCT Compound embedded RFP-GFP-LC3 hearts were transversely sectioned by cryotome (8–12 μm thickness) at the mid-portion of the ventricles, mounted in ProLong Diamond Antifade (with DAPI, Life Technologies, Carlsbad, CA, United States, #P36962), and dried in the dark at room temperature for 24 h. On an Olympus FLUOVIEW FV5OO/IX81 confocal laser scanning microscope at ×60 oil magnification, cardiac muscles were analyzed for the dual LC3-RFP-GFP reporters. Excitation was set at 405 nm (DAPI), 488 nm (GFP), and 543 nm (RFP). The LC3 Tg + mouse strain allowed for tracking of the dual tagged RFP (red fluorescent protein)—GFP (green fluorescent protein)—LC3 protein, allowing for identification of different autophagic phases. As the LC3 protein starts to group up to form early phase autophagosomes, red (RFP) and green (GFP) puncta together appear yellow under a confocal fluorescent microscope. As the autophagosome fuses with a lysosome to form a late phase autolysosome, the acidic hydrolases from the lysosome quench the less stable GFP (green) signal, leaving behind only the RFP (red) signal. Images were quantified in five to ten random fields (representing all walls of the ventricles) from each animal and then analyzed using ImageJ algorithm (Pampliega et al., 2013).

### Protein expression

Approximately, 25–35 mg of cardiac and skeletal muscle tissue was homogenized *via* 8 M urea lysis buffer with a TissueLyser LT homogenizer (Hilden, Germany). Homogenates were centrifuged for 10 min at 5000 rpm at 4 °C, and the supernatant was collected for protein analysis. Bradford method was used to determine protein concentration. Protein (25–30 μg) was loaded onto 4–12% Bis-Tris gels and separated by NuPAGE MES running buffer (Thermofisher, MA, United States ) for 30–40min at constant 200 V. Proteins were transferred to PVDF membranes for 60min at 30 V. PVDF membranes were blocked in 5% nonfat milk for an hour at room temperature, and then incubated with primary antibody diluted in 5% milk (anti-P-NF-κβ p65, 1:200 dilution [CST3033s]; anti-MyD88, 1:1000 dilution [CST4283S]; anti-Beclin-1, 1:1000 dilution [CST3495]; anti-pFOXO1/3a, 1:500 dilution [CST 9464S]; anti-FOXO1, 1:500 dilution [CST 2880S]) at 4 °C overnight. Membranes were then incubated with species-specific secondary antibodies diluted in 5% milk (rabbit, 1:5000 [Sigma A9169]; mouse, 1:10,000 [Sigma A2228]) for 1 hour at room temperature. Membranes were developed using ECL Select (GE Healthcare, RPN2235), imaged using a Bio-Rad Chem-Doc XRS+ (Hercules, CA, United States ), and band intensity was quantified using Quantity One (Bio Rad; Hercules, CA, United States ). Membranes were probed for the loading control protein anti-GAPDH (1:4000 [Sigma 039M4772 V] in 5% milk) overnight at 4 °C and then incubated with secondary antibody (mouse; 1:10,000, diluted in 5% milk) for 1 hour at room temperature. Membranes were developed in ECL, imaged and band intensity was quantified using Quantity One. Protein concentrations were determined by means of densitometry (Bio-Rad, Hercules, CA, United States ) and normalized to the loading control GAPDH.

### Statistical analysis

All data are presented as mean ± standard deviation (SD). All statistics were performed using the statistical software GraphPad Prism (La Jolla, CA, United States ). To determine differences between groups, a one-way ANOVA was performed for each variable. Two-way repeated measures ANOVA was performed to compare baseline, post-exercise, and sacrifice measurements of select variables. Change score measurements were performed to calculate percent changes from baseline to sacrifice for select variables. Differences in tumor characteristics were analyzed *via* a Student’s *t*-test. All analyses were two-tailed and an alpha level of 0.05 was used to define statistical significance. For ANOVAs, if a significant difference (*p* < 0.05) was identified, Tukey’s post hoc testing was performed to identify where the significant differences occurred.

## Results

### Effect of preconditioning exercise and tumor burden on body and cardiac muscle mass

**FIGURE 1:**
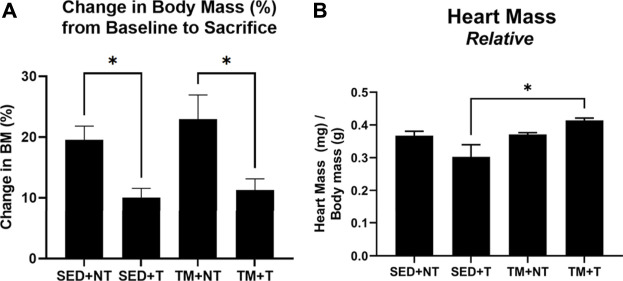
Change in Body Mass Overtime and Relative Cardiac Mass at Sacrifice. Tumor bearing lowered body mass **(A)**. SED + T group exhibited smallest relative cardiac mass compared to all other groups and was significantly smaller than the TM + T group **(B)**. SED, sedentary; TM, preconditioning treadmill endurance exercise training; T, tumor bearing; non-tumor. Data are presented as mean ± SD. *p < 0.05.

Mice were trained in the form of preconditioning endurance treadmill exercise or remained sedentary for 8 weeks. After the 8-week exercise protocol, animals assigned to the tumor bearing groups were inoculated in the left flank with Lewis Lung Carcinoma cells to grow localized tumors allowing for convenient growth measurement and morphology determination. Tumor development was monitored for an additional 4 weeks. At the end of the 12-week protocol, body weight was measured, hearts were excised, wet cardiac muscle mass was measured and normalized to body weight. No differences were observed in body mass at baseline and at the end of the 8-week training period. As summarized in Figure 1, both tumor bearing groups exhibited significantly smaller change increases in body mass overtime compared to the non-tumor bearing groups (*p* < 0.05). Sedentary tumor bearing mice (SED + T) exhibited the smallest relative cardiac muscle mass compared to all other groups and was significantly smaller than the exercise preconditioned tumor bearing group (TM + T). These data indicate that tumor burden alone can negatively impact body and muscle mass, resulting in cardiac atrophy. The data also shows that exercise prior to tumor burden can attenuate these effects.

### Effects of exercise on tumor growth

**FIGURE 2:**
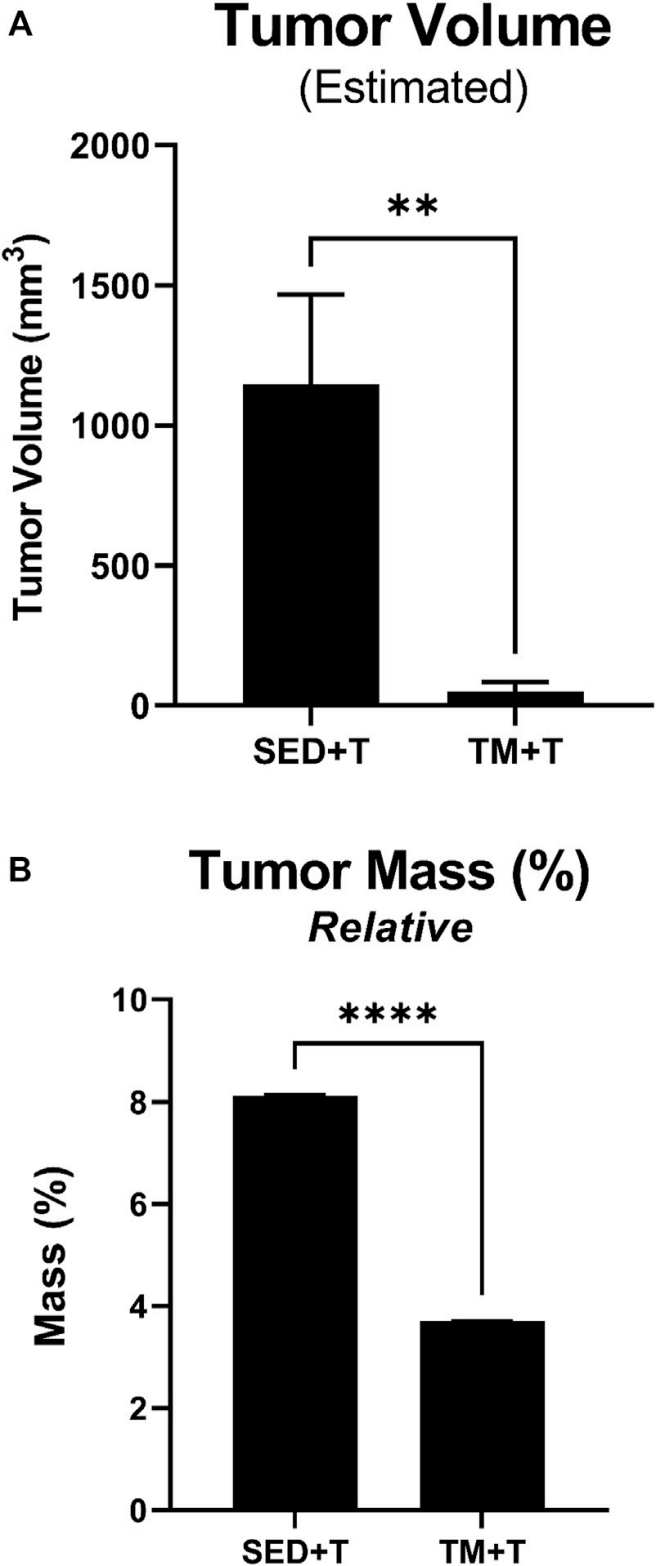
Tumor Mass and Volume. Preconditioning endurance exercise was associated with a significantly reduced estimated tumor volume **(A)** and relative tumor mass **(B)** based on length, width, and thickness tumor measurements. SED, sedentary; TM, preconditioning treadmill endurance exercise training; T, tumor bearing. Data are presented as mean ± SD. **p < 0.01, ****p < 0.001.

During the 4-week tumor bearing period post exercise, tumor development and body weight changes were monitored, morphology was assessed, and tumor growth measurements were taken. Final measurements were taken at the endpoint of the study and used to calculate estimated tumor volume and tumor mass relative to body weight (Figure 2). Tumor evaluation data, as summarized in Figure 2, showed a 22-fold decrease in estimated tumor volume (*p* < 0.05) in the preconditioning treadmill trained group compared to the sedentary tumor bearing group (Figure 2A). Additionally, analysis of relative tumor mass indicated a 45% decrease in tumor mass normalized to body weight (*p* < 0.05) in the TM + T mice compared to sedentary tumors (Figure 2B).

### Effects of exercise and tumor burden on cardiac function

**FIGURE 3:**
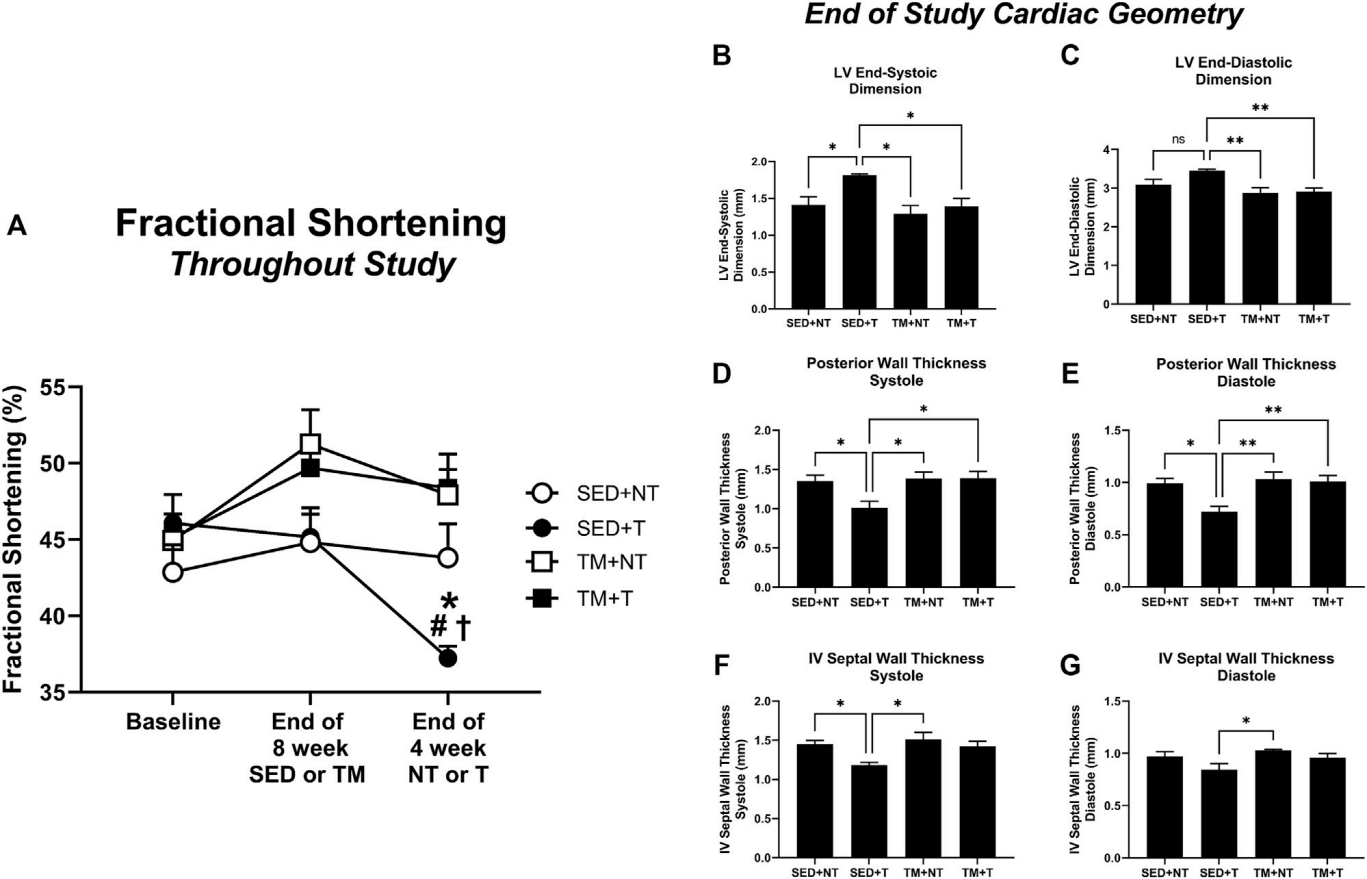
Cardiac Function. Tumor bearing results in a significant decline in cardiac function, thinning of walls, and enlarged left ventricle chamber size. Preconditioning endurance treadmill exercise preserves cardiac function in tumor-bearing animals based on fractional shortening analysis. SED, sedentary; TM, preconditioning treadmill endurance exercise training; T, tumor bearing; NT, non-tumor. Data are presented as mean ± SD. **(A)** *p < 0.05 vs SED + NT, ^#^ *p* < 0.05 vs TM + NT, ^†^ *p* < 0.05 vs TM + T. **(B–G)**, *p < 0.05, **p < 0.01.

Cardiac function was tracked throughout the study *via* conscious echocardiography. No differences were detected between groups at the beginning of the study (Figure 3A, baseline). While cardiac function as measured by fractional shortening improved in treadmill trained mice, these improvements were not statistically significant (Figure 3A, end of 8-week SED or TM). However, 4 weeks of tumor bearing elicited a significant decline in cardiac function in sedentary mice (SED + T). SED + T mice exhibited thinning of ventricular walls during systole and diastole (Figure 3D–G), enlarged ventricles during systole and diastole (Figure 3B, C), and significantly reduced fractional shortening (Figure 3A). Our data suggest that tumor bearing can cause cardiac dysfunction that is consistent with a dilated cardiomyopathy phenotype. Interestingly, cardiac dysfunction was not observed in the treadmill trained tumor bearing mice (TM + T). Therefore, 8 weeks of progressive treadmill training *prior to* tumor bearing appears to preserve cardiac function in the face of tumor mediated cardiac challenges.

### Effects of exercise and tumor burden on cardiac autophagy

**FIGURE 4:**
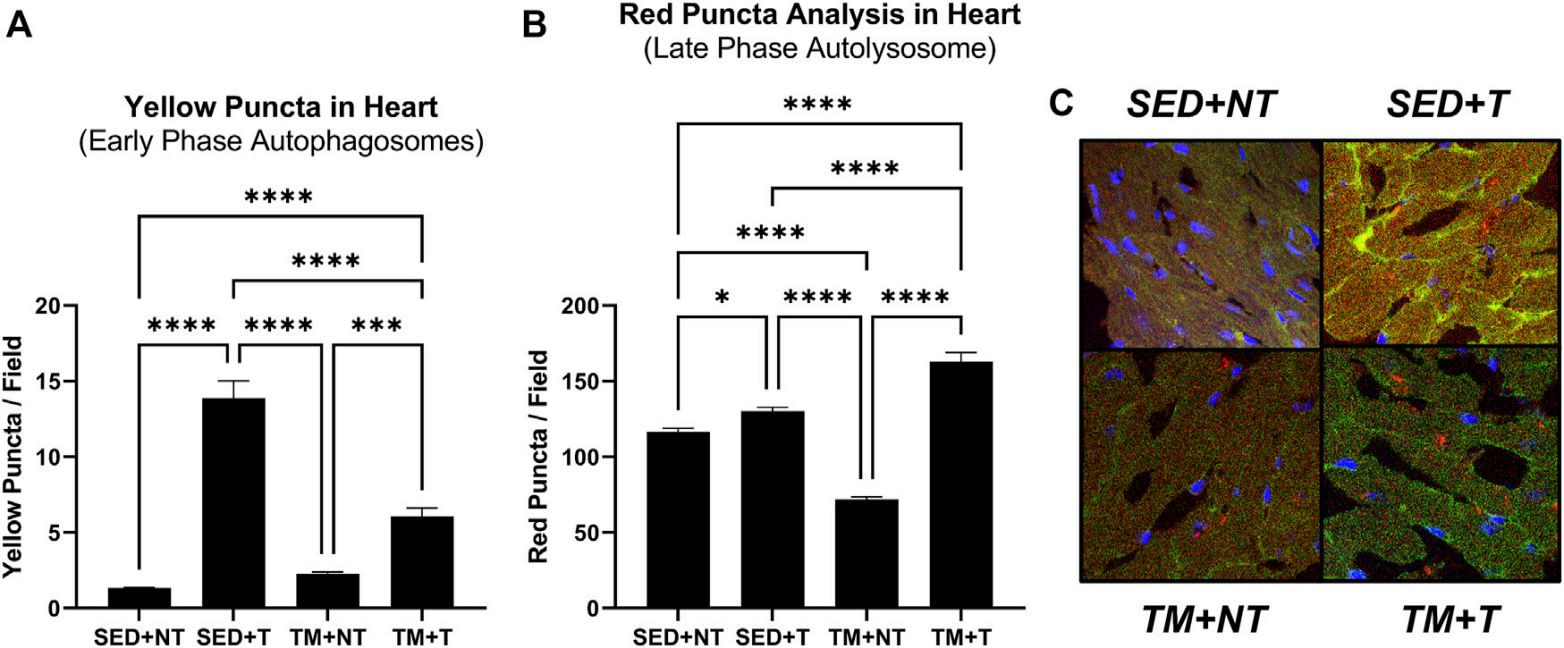
Cardiac Autophagy. Tumor bearing significantly increases early phase **(A)** and late phase autophagy **(B)**. Representative images **(C)**. Preconditioning treadmill endurance exercise modulates early phase autophagy **(A)**. SED, sedentary; TM, preconditioning treadmill endurance exercise training; T, tumor bearing; NT, non-tumor. Data are presented as mean ± SD. *p < 0.05, **p < 0.01, ****p < 0.001.

The expression of cardiac autophagy was analyzed to further investigate how tumor burden may negatively affect cardiac function, specifically analyzing the effect of tumor burden and exercise on early and late phase autophagy. Autophagy was analyzed *via* fluorescent microscopy identifying different conformational stages of the dual LC3-GFP-RFP reporters. Early phase autophagosomes appear yellow (GFP + RFP = yellow puncta) while late phase autolysosomes appear red (GFP signal gets quenched leaving behind only RFP = red puncta). Animals from both tumor bearing groups (SED + T; TM + T) expressed significantly more early phase autophagosomes (yellow puncta, *p* < 0.05) (Figure 4A) and late phase autolysosomes (red puncta, *p* < 0.05) (Figure 4B) compared to the non-tumor bearing groups (NT). Mice that underwent preconditioning treadmill training *prior* to tumor burden (TM + T) expressed significantly less early phase autophagosomes (yellow puncta, *p* < 0.05) compared to SED + T mice, yet significantly more late phase autolysosomes (red puncta, *p* < 0.05). Together, this data indicates that while exercise preconditioning may help modulate cardiac autophagy to protect against tumor-mediated increases in early phase autophagosomes but not late phase autolysosomes.

### Effects of exercise and tumor burden on cellular protein expression

**FIGURE 5:**
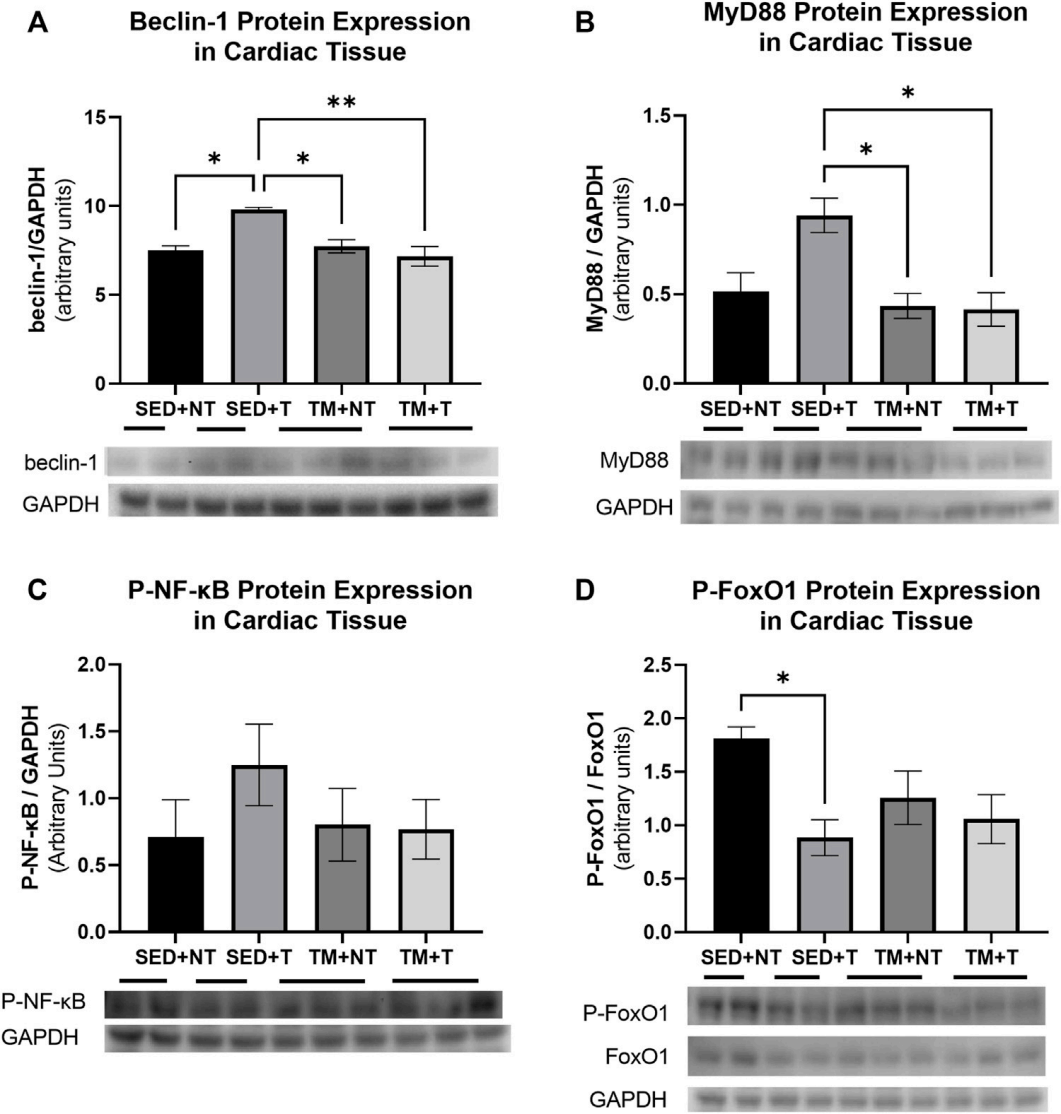
Cardiac expression of autophagy protein Beclin1 **(A)**; inflammatory markers MyD88 **(B)** and p-NF-kB **(C)**; and atrophy protein p-FOXO1 **(D)**. Tumor bearing results in increased autophagy (belcin-1), inflammation (MyD88), and atrophy and metabolism (p-FOXO1). Preconditioning exercise abolished these responses. SED, sedentary; TM, preconditioning treadmill endurance exercise training; T, tumor bearing; NT, non-tumor. Data are presented as mean ± SD. *p < 0.05, **p < 0.01.

The present study sought to determine if the observed cardiac dysfunction might be attributed to enhanced inflammation, dysfunctional metabolism, or upregulated autophagic processes. Therefore, homogenates of the apex of the heart were analyzed for the expression of the autophagy protein beclin-1, the metabolic and atrophy protein p-FOXO1, and pro-inflammatory markers MyD88 (IL-1β signaling) and p-NF-κβ, to further investigate the cellular mechanisms of how tumor burden may negatively affect cardiac structure and function (Figure 5). Beclin-1 interacts with the autophagosome to initiate early phase autophagy. As summarized in Figure 5A, SED + T mice exhibited significantly more beclin-1 expression compared to all other groups indicating that autophagy was upregulated in SED + T cardiac muscle. TM + T mice expressed significantly less beclin-1 compared to the SED + T control group, indicating that treadmill training at least partially downregulated autophagic processes, suggesting cardioprotective effects induced by preconditioning exercise. This is one possible mechanism contributing to the differences in cardiac function between groups. Inflammatory IL-1β (MyD88) and NF-κβ signaling pathways were upregulated in SED + T compared to all other groups indicating inflammatory pathways likely play a role in the cancer-mediated cardiac dysfunction. Specifically, MyD88 (IL-1β) was significantly increased compared the TM + T group which may indicate preconditioned exercise is able to protect against tumor-mediated cardiac inflammation that may serve to protect the heart. Finally, the atrophy and metabolism associated protein (inactive) p-FOXO1 was downregulated in SED + T group compared to SED + NT group, indicating tumor-bearing may cause cardiac metabolism abnormalities and atrophy. Importantly, the TM + T group was not significantly different than SED + NT indicating that preconditioned exercise may protect against tumor-mediated metabolic perturbations and atrophy.

## Discussion

Since the heart is unable to regenerate, autophagy is an important bulk degradation and turnover mechanism in the heart. Under normal conditions, basal levels of autophagy are critical to cardiac metabolism and function. However, under conditions of stress, such as starvation, autophagy can become exceedingly elevated and promote harmful cardiac remodeling (Nishida et al., 2009). Our research suggests that tumor bearing is capable of eliciting cardiac dysfunction consistent with a dilated cardiomyopathy phenotype and cancer cachexia. This may be related to the enhanced levels of autophagy observed during tumor bearing. Elevated autophagy has been associated with dilated cardiomyopathy (Kanamori et al., 2022), and autophagy and necrosis associated with dilated cardiomyopathy can lead to adverse cardiac remodeling (Nishida et al., 2009). Autophagic vacuole quantification has been shown to predict death or hospital readmission due to heart failure recurrence (Saito et al., 2016).

In our mouse tumor model, sedentary tumor bearing mice (SED + T) showed elevated levels of early phase autophagosomes and late phase autolysosomes compared to sedentary non-tumor controls. This elevated autophagy coincided with a significant increase in beclin-1 protein levels. Beclin-1 and Vps34 are critical initiators of autophagy (Kihara et al., 2001; Maejima et al., 2016). Unsurprisingly, cancer cachexia patients show elevated levels of beclin-1 as well as increased levels of the autophagy marker protein, LC3B-II (Aversa et al., 2016). Furthermore, cachectic Apc (min/+) mice show elevated levels of beclin-1, significantly smaller hearts than healthy controls, and decreased mTOR signaling and protein synthesis (Manne et al., 2013). In a mouse model of pulmonary hypertension, elevated levels of beclin-1 and autophagy (LC3-II protein levels and autophagic vacuoles) coincided with significantly worse cardiac function (Deng et al., 2017).

Importantly, both inflammation and FOXO1 signaling have been implicated in increased autophagy in the failing heart. During cardiac glucose deprivation, FOXO1 and FOXO3a transcriptionally upregulate autophagy genes to promote cardiac autophagy which coincides with smaller cardiomyocyte size (Sengupta et al., 2009). When inflammation and FOXO1 activity are upregulated (i.e., increased MyD88 and decreased inactive p-FOXO1 as observed in the present study), the heart showed negative remodeling resulting in failure (Rubio et al., 2021). Together, this data help explain the observed tumor-mediated cardiac dysfunction that is likely related to an upregulation in cardiac inflammation (MyD88) and autophagy (beclin-1) leading to metabolic perturbations and atrophy (p-FOXO1).

Exercise is well known to be cardioprotective (Quindry et al., 2007; Kavazis et al., 2008; Powers et al., 2014; Parry et al., 2018). More recently, exercise preconditioning–exercise initiated prior to stimuli or intervention–has been shown to have lasting beneficial effects, even after exercise has been ceased (Hydock et al., 2011; Thijssen et al., 2018; Quindry and Franklin, 2021). This includes protection against ischemia/reperfusion injury (Quindry and Hamilton, 2013) and reduces risk of cardiovascular events (Thijssen et al., 2018), and can include mechanisms such as improved oxidant buffering and mitochondrial protection (Frasier et al., 2011). More recently, studies have shown that exercise preconditioning might confer at least part of its cardioprotection through changes in cardiac autophagy (elevated LC3-II, Atg7, Atg4B, and Atg3 protein levels). In this study, it was noted that early stages of exercise preconditioning promote greater levels of autophagy than later stages of exercise preconditioning (Wan et al., 2021). Exercise preconditioning is also known to reduce inflammation-mediated cardiac injury in rats (Li et al., 2020). Finally, 7 days of exercise preconditioning was able to downregulate FOXO1 mediated atrogenes in the gastrocnemius of mice, and it was surmised that a longer, more intense exercise preconditioning regimen (as observed in the present study) may suppress atrogenes and thus protect the muscle for a longer period of time (Brocca et al., 2021). Therefore, there may be cardioprotective value in exercise preconditioning.

Studies have shown that exercise is capable of modulating autophagy in a helpful manner, essentially keeping it at basal to slightly elevated levels to keep the tissue healthy (He et al., 2012; Jamart et al., 2012; Martin-Rincon et al., 2018). In a model of cancer cachexia, muscle autophagy was significantly elevated, and this coincided with smaller muscles, poor muscle morphology, and increased fatigue levels. However, voluntary wheel running was able to modulate autophagy to restore it to basal levels. This coincided with preserving muscle mass, muscle morphology, and improved (lessened) fatigue (Pigna et al., 2016). This agrees with findings from a Mat-B-III tumor model where tumor bearing induced significant increases in cardiac autophagy (LC3-II protein levels), a significant αMHC to βMHC cardiac isoform shift, and worse cardiac function. However, voluntary wheel running was able to counter tumor-mediated levels of autophagy, preserve cardiac myosin heavy chain isoform ratios, and preserved cardiac function (Parry and Hayward, 2018). Taken together, it is possible that exercise preconditioning modulates cardiac autophagy in a way that counters tumor-mediated dysfunctional autophagy to protect against tumor-mediated cardiac dysfunction.

Interestingly, our study also showed that exercise *preconditioning* severely stunted tumor growth. One study in B16-F10 melanoma implantation showed that exercise preconditioning resulted in slower tumor cell growth compared to exercise after tumor cell implantation (Pedersen et al., 2016). Significantly less research has been conducted in this area, yet it remains an important effector of overall health. Given the effectiveness of preventative health for reducing healthcare costs, determining the underlying beneficial mechanisms of exercise should remain a priority.

## Conclusion

Our research indicates that tumor bearing can elicit a dilated cardiomyopathy phenotype associated with increased levels of autophagy. Enhanced levels of autophagy may be related to a tumor-mediated upregulation of cardiac beclin-1 expression and cardiac inflammation (MyD88), which lead to increased cardiac atrophy (FOXO1 signaling). Exercise preconditioning–exercise completed prior to tumor bearing–appears to have lasting benefits capable of protecting the heart from tumor-mediated cardiac dysfunction (even if exercise is not continued during the tumor bearing phase). Exercise may serve to beneficially modulate cardiac autophagy *via* beclin-1 to maintain advantageous levels of autophagy. Finally, exercise completed *prior* to tumor bearing severely stunted tumor growth. It is possible that this reduced tumor burden contributes to the cardioprotective effects of exercise preconditioning. Therefore, exercise appears to offer multiple lasting benefits, even after it has been ceased. Such evidence is critical to understanding the health advantages of exercise and physical activity lifestyle modifications. This study highlights the importance of a physically active lifestyle in reducing risk, incidence, and severity of chronic diseases like cancer.

## Data Availability

The raw data supporting the conclusion of this article will be made available by the authors, without undue reservation.

## References

[B1] Argiles J. M. Busquets S. Stemmler B. Lopez-Soriano F. J. (2014). Cancer cachexia: understanding the molecular basis. Nat. Rev. Cancer 14 (11), 754–762. 10.1038/nrc3829 25291291

[B2] Aversa Z. Pin F. Lucia S. Penna F. Verzaro R. Fazi M. (2016). Autophagy is induced in the skeletal muscle of cachectic cancer patients. Sci. Rep. 6 (1), 30340–30411. 10.1038/srep30340 27459917 PMC4962093

[B3] Brocca L. Rossi M. Canepari M. Bottinelli R. Pellegrino M. A. (2021). Exercise preconditioning blunts early atrogenes expression and atrophy in gastrocnemius muscle of hindlimb unloaded mice. Int. J. Mol. Sci. 23 (1), 148. 10.3390/ijms23010148 35008572 PMC8745338

[B4] Couch M. E. Dittus K. Toth M. J. Willis M. S. Guttridge D. C. George J. R. (2015). Cancer cachexia update in head and neck cancer: definitions and diagnostic features. Head. Neck 37 (4), 594–604. 10.1002/hed.23599 24415363

[B5] Deng Y. Wu W. Guo S. Chen Y. Liu C. Gao X. (2017). Altered mTOR and Beclin-1 mediated autophagic activation during right ventricular remodeling in monocrotaline-induced pulmonary hypertension. Respir. Res. 18 (1), 53–15. 10.1186/s12931-017-0536-7 28340591 PMC5366117

[B6] Fearon K. Strasser F. Anker S. D. Bosaeus I. Bruera E. Fainsinger R. L. (2011). Definition and classification of cancer cachexia: an international consensus. Lancet. Oncol. 12 (5), 489–495. 10.1016/s1470-2045(10)70218-7 21296615

[B7] Frasier C. R. Moore R. L. Brown D. A. (2011). Exercise-induced cardiac preconditioning: how exercise protects your achy-breaky heart. J. Appl. Physiol. 111 (3), 905–915. 10.1152/japplphysiol.00004.2011 21393468

[B8] He C. Bassik M. C. Moresi V. Sun K. Wei Y. Zou Z. (2012). Exercise-induced BCL2-regulated autophagy is required for muscle glucose homeostasis. Nature 481 (7382), 511–515. 10.1038/nature10758 22258505 PMC3518436

[B9] Hydock D. S. Lien C. Y. Jensen B. T. Schneider C. M. Hayward R. (2011). Exercise preconditioning provides long-term protection against early chronic doxorubicin cardiotoxicity. Integr. Cancer Ther. 10 (1), 47–57. 10.1177/1534735410392577 21382960

[B10] Jamart C. Francaux M. Millet G. Y. Deldicque L. Frère D. Féasson L. (2012). Modulation of autophagy and ubiquitin-proteasome pathways during ultra-endurance running. J. Appl. Physiol. 112 (9), 1529–1537. 10.1152/japplphysiol.00952.2011 22345427

[B11] Kanamori H. Yoshida A. Naruse G. Endo S. Minatoguchi S. Watanabe T. (2022). Impact of autophagy on prognosis of patients with dilated cardiomyopathy. J. Am. Coll. Cardiol. 79(8), 789–801. 10.1016/j.jacc.2021.11.059 35210034

[B12] Kavazis A. N. McClung J. M. Hood D. A. Powers S. K. (2008). Exercise induces a cardiac mitochondrial phenotype that resists apoptotic stimuli. Am. J. Physiol. Heart Circ. Physiol. 294 (2), H928–H935. 10.1152/ajpheart.01231.2007 18083894

[B13] Kazemi-Bajestani S. M. Becher H. Fassbender K. Chu Q. Baracos V. E. (2014). Concurrent evolution of cancer cachexia and heart failure: bilateral effects exist. J. Cachexia Sarcopenia Muscle 5 (2), 95–104. 10.1007/s13539-014-0137-y 24627226 PMC4053562

[B14] Kihara A. Noda T. Ishihara N. Ohsumi Y. (2001). Two distinct Vps34 phosphatidylinositol 3–kinase complexes function in autophagy and carboxypeptidase Y sorting in *Saccharomyces cerevisiae* . J. Cell Biol. 152 (3), 519–530. 10.1083/jcb.152.3.519 11157979 PMC2196002

[B15] Kumar N. B. Kazi A. Smith T. Crocker T. Yu D. Reich R. R. (2010). Cancer cachexia: traditional therapies and novel molecular mechanism-based approaches to treatment. Curr. Treat. Options Oncol. 11 (3-4), 107–117. 10.1007/s11864-010-0127-z 21128029 PMC3016925

[B16] Li Y. Xu P. Wang Y. Zhang J. Yang M. Chang Y. (2020). Different intensity exercise preconditions affect cardiac function of exhausted rats through regulating TXNIP/TRX/NF-ĸB(p65)/NLRP3 inflammatory pathways. Evid. Based. Complement. Altern. Med. 2020, 5809298. 10.1155/2020/5809298 PMC7301185 32595731

[B17] Maejima Y. Isobe M. Sadoshima J. (2016). Regulation of autophagy by Beclin 1 in the heart. J. Mol. Cell. Cardiol. 95, 19–25. 10.1016/j.yjmcc.2015.10.032 26546165 PMC4861696

[B18] Manne N. D. P. K. Lima M. Enos R. T. Wehner P. Carson J. A. Blough E. (2013). Altered cardiac muscle mTOR regulation during the progression of cancer cachexia in the ApcMin/+ mouse. Int. J. Oncol. 42 (6), 2134–2140. 10.3892/ijo.2013.1893 23589074 PMC3699594

[B19] Martin‐Rincon M. Morales‐Alamo D. Calbet J. (2018). Exercise‐mediated modulation of autophagy in skeletal muscle. Scand. J. Med. Sci. Sports 28 (3), 772–781. 10.1111/sms.12945 28685860

[B20] Murphy K. T. (2016). The pathogenesis and treatment of cardiac atrophy in cancer cachexia. Am. J. Physiol. Heart Circ. Physiol. 310 (4), H466–H477. 10.1152/ajpheart.00720.2015 26718971

[B21] Nilsson M. I. Bourgeois J. M. Nederveen J. P. Leite M. R. Hettinga B. P. Bujak A. L. (2019). Lifelong aerobic exercise protects against inflammaging and cancer. PloS one 14 (1), e0210863. 10.1371/journal.pone.0210863 30682077 PMC6347267

[B22] Nishida K. Kyoi S. Yamaguchi O. Sadoshima J. Otsu K. (2009). The role of autophagy in the heart. Cell Death Differ. 16 (1), 31–38. 10.1038/cdd.2008.163 19008922

[B23] Pampliega O. Orhon I. Patel B. Sridhar S. Díaz-Carretero A. Beau I. (2013). Functional interaction between autophagy and ciliogenesis. Nature 502 (7470), 194–200. 10.1038/nature12639 24089209 PMC3896125

[B24] Parry T. L. Hayward R. (2018). Exercise protects against cancer-induced cardiac cachexia. Med. Sci. Sports Exerc. 50 (6), 1169–1176. 10.1249/mss.0000000000001544 29315166

[B25] Parry T. L. Starnes J. W. O'Neal S. K. Bain J. R. Muehlbauer M. J. Honcoop A. (2018). Untargeted metabolomics analysis of ischemia-reperfusion-injured hearts *ex vivo* from sedentary and exercise-trained rats. Metabolomics 14 (1), 8. 10.1007/s11306-017-1303-y 30104954 PMC6086497

[B26] Parry T. L. Willis M. S. (2016). Cardiac ubiquitin ligases: Their role in cardiac metabolism, autophagy, cardioprotection and therapeutic potential. Biochim. Biophys. Acta 1862 (12), 2259–2269. 10.1016/j.bbadis.2016.07.002 27421947 PMC5159290

[B27] Pedersen L. Idorn M. Olofsson G. H. Lauenborg B. Nookaew I. Hansen R. H. (2016). Voluntary running suppresses tumor growth through epinephrine-and IL-6-dependent NK cell mobilization and redistribution. Cell Metab. 23 (3), 554–562. 10.1016/j.cmet.2016.01.011 26895752

[B28] Penna F. Costamagna D. Pin F. Camperi A. Fanzani A. Chiarpotto E. M. (2013). Autophagic degradation contributes to muscle wasting in cancer cachexia. Am. J. Pathol. 182 (4), 1367–1378. 10.1016/j.ajpath.2012.12.023 23395093

[B29] Pigna E. Berardi E. Aulino P. Rizzuto E. Zampieri S. Carraro U. (2016). Aerobic exercise and pharmacological treatments counteract cachexia by modulating autophagy in colon cancer. Sci. Rep. 6 (1), 26991–27014. 10.1038/srep26991 27244599 PMC4886631

[B30] Powers S. K. Smuder A. J. Kavazis A. N. Quindry J. C. (2014). Mechanisms of exercise-induced cardioprotection. Physiology 29 (1), 27–38. 10.1152/physiol.00030.2013 24382869 PMC3929117

[B31] Quindry J. C. Franklin B. A. (2021). Exercise preconditioning as a cardioprotective phenotype. Am. J. Cardiol. 148, 8–15. 10.1016/j.amjcard.2021.02.030 33675772

[B32] Quindry J. C. Hamilton K. L. French J. P. Lee Y. Murlasits Z. Tumer N. (2007). Exercise-induced HSP-72 elevation and cardioprotection against infarct and apoptosis. J. Appl. Physiol. 103 (3), 1056–1062. 10.1152/japplphysiol.00263.2007 17569768

[B33] Quindry J. Hamilton K. (2013). Exercise and cardiac preconditioning against ischemia reperfusion injury. Curr. Cardiol. Rev. 9 (3), 220–229. 10.2174/1573403x113099990033 23909636 PMC3780347

[B34] Rubio B. Mora C. Pintado C. Mazuecos L. Fernández A. López V. (2021). The nutrient sensing pathways FoxO1/3 and mTOR in the heart are coordinately regulated by central leptin through PPARβ/δ. Implications in cardiac remodeling. Metabolism. 115, 154453. 10.1016/j.metabol.2020.154453 33249043

[B35] Saito T. Asai K. Sato S. Hayashi M. Adachi A. Sasaki Y. (2016). Autophagic vacuoles in cardiomyocytes of dilated cardiomyopathy with initially decompensated heart failure predict improved prognosis. Autophagy 12 (3), 579–587. 10.1080/15548627.2016.1145326 26890610 PMC4836017

[B36] Sandri M. (2013). Protein breakdown in muscle wasting: role of autophagy-lysosome and ubiquitin-proteasome. Int. J. Biochem. Cell Biol. 45 (10), 2121–2129. 10.1016/j.biocel.2013.04.023 23665154 PMC3775123

[B37] Sengupta A. Molkentin J. D. Yutzey K. E. (2009). FoxO transcription factors promote autophagy in cardiomyocytes. J. Biol. Chem. 284 (41), 28319–28331. 10.1074/jbc.M109.024406 19696026 PMC2788882

[B38] Thijssen D. H. Redington A. George K. P. Hopman M. T. Jones H. (2018). Association of exercise preconditioning with immediate cardioprotection: a review. JAMA Cardiol. 3 (2), 169–176. 10.1001/jamacardio.2017.4495 29188285

[B39] Wan D.-F. Pan S.-S. Tong Y.-S. Huang Y. (2021). Exercise preconditioning promotes autophagy to cooperate for cardioprotection by increasing LC3 lipidation-associated proteins. Front. Physiol. 12, 599892. 10.3389/fphys.2021.599892 34025444 PMC8131968

[B40] Zhao M. Veeranki S. P. Magnussen C. G. Xi B. (2020). Recommended physical activity and all cause and cause specific mortality in US adults: prospective cohort study. Bmj 370, m2031. 10.1136/bmj.m2031 32611588 PMC7328465

